# Action prediction modulates both neurophysiological and psychophysical indices of sensory attenuation

**DOI:** 10.3389/fnhum.2014.00115

**Published:** 2014-02-28

**Authors:** Cedric Roussel, Gethin Hughes, Florian Waszak

**Affiliations:** ^1^Laboratoire de Psychologie de la Perception, Université Paris Descartes, Sorbonne Paris CitéParis, France; ^2^Laboratoire Psychologie de la Perception, Centre National de la Recherche Scientifique-Université Paris Descartes, UMR 8158Paris, France; ^3^Department of Psychology, University of EssexColchester, UK

**Keywords:** action prediction, effect prediction, psychophysycs, neurophysiology, contrast discrimination

## Abstract

Sensory attenuation refers to the observation that stimuli that are predicted based on one’s action are attenuated. This phenomenon has primarily been observed as a neurophysiological phenomenon, with reduced Event-Related Potential (ERP) (e.g., Bäss et al., 2008) and BOLD (e.g., Blakemore et al., 1998). However, psychophysical investigations (e.g., Sato, 2008; Cardoso-Leite et al., 2010; Roussel et al., 2013) have confirmed that action prediction also influences the perception of sensory action effects. The present study recorded both neurophysiological and psychophysical measures in a single experiment, to confirm whether the two phenomena are related. In addition, by measuring the ERP modulations of both stimulus contrast and prediction congruency, we sought to directly relate the neurophysiological phenomenon to the magnitude of sensory processing in the brain. Participants performed left- and right-hand voluntary actions that were previously associated with the letters A and H. In the test phase, participants were presented with these same two letters, at one of two possible contrasts. Participants were required to report which of the two possible contrasts had been presented. We observed both reduced contrast discrimination (in line with Roussel et al., 2013) and a reduced ERP response for congruent action-effects. Furthermore, our congruency modulation was observed on the same component that differed as a function of stimulus contrast. Taken together these results strongly suggest that neurophysiological indices of sensory attenuation reflect reduced sensory processing of voluntary action effects.

## Introduction

The ability to produce intended effects in the environment is an important aspect of action control. The ideomotor theory of action claims that bidirectional associations are formed between an action’s motor code and the ensuing sensory effects. These associations can be used to select an action by anticipating or internally activating their perceptual consequences (e.g., Greenwald, 1970; Prinz, 1997; Elsner and Hommel, 2001; Herwig et al., 2007; Waszak et al., 2012). A similar approach has also been employed in the development of forward models of action, which claim that an efference copy, generated during action selection, is used to predict future behavioral state of the system as well as the sensory consequences of that behavior (Wolpert et al., 1995; Wolpert and Miall, 1996). Many computational models also include inverse models that provide the motor command which, given the particular current state, would result in a desired end state, for example, a particular sensory effect (Wolpert et al., 1995).

These principles of action prediction have been investigated using a number of different methodologies. One line of evidence comes from paradigms studying processing of anticipated action effects. Notably, it has been shown that self-generated stimuli are perceived as less intense than externally generated stimuli, a phenomenon known as sensory attenuation. Sensory attenuation has been demonstrated in the somatosensory (Blakemore et al., 1998), the auditory (Sato, 2008) and the visual domain (Cardoso-Leite et al., 2010; Roussel et al., 2013). Cardoso-Leite et al. (2010), for example, studied the influence of the congruency between anticipated and actual action effect (tilted Gabor stimuli) on the detection of the latter. Detection performance in the congruent condition (i.e., when the effect anticipated by the action and the true effect matched) was worse than in the neutral and incongruent conditions, demonstrating sensory attenuation. Studies investigating sensory attenuation as a perceptual phenomenon have been complemented by studies investigating neurophysiological correlates of anticipated action effects (e.g., Schafer and Marcus, 1973; Blakemore et al., 1998; Bäss et al., 2008; Aliu et al., 2009; Gentsch and Schütz-Bosbach, 2011; Hughes and Waszak, 2011; Hughes et al., 2013b). To give an example, Bäss et al. (2008) found a reduced auditory N1 component for action-triggered tones compared to externally triggered tones, suggesting that cortical activity was attenuated for the former.

Both attenuated phenomenological and neurophysiological responses are usually interpreted along the same lines in terms of forward models (e.g., Miall and Wolpert, 1996), as if they reflect the same mechanism. At the same time, this mechanism has usually been considered to be relatively low-level (“sensory”). However, a systematic investigation of the relationship between sensory attenuation as a perceptual phenomenon, on the one side, and as a neurophysiological phenomenon, on the other side, is missing. As a consequence, it is impossible to tell how the attenuation of perceptual awareness is related to the attenuation of cortical responses that have been observed a number of times in separate experiments. Moreover, concerning the locus of the effect, differences in cortical responses between conditions with and without effect anticipation cannot always be unequivocally attributed to sensory processing. Often they may also be caused by other differences in attentional and cognitive processing (cf., Waszak et al., 2012; Hughes et al., 2013a). As a consequence, different studies do not always converge to the same conclusions. For example, Bäss et al. (2008) observed attenuated fronto-central negativity when comparing action-triggered vs. externally triggered auditory stimuli. They concluded that early sensory processing in the auditory cortex is reduced. By contrast, Hughes and Waszak (2011) compared Event-Related Potential (ERPs) to action-triggered vs. externally triggered visual stimuli. They observed an increased, not a decreased, visual P1 component. In this study, attenuated cortical responses were observed in a frontoparietal network, starting 150 ms after stimulus. This result would be in line with the findings of Del Cul et al. (2007) showing that subjective thresholds of visual stimuli is reflected in later processing in a fronto-parietal network, rather than in early visual areas.

The aim of the current experiment was to shed new light on two interrelated questions. First, we investigated whether neurophysiological indices of sensory attenuation reflect early, low-level or later, higher-level mechanisms. Second, we explored how neurophysiological and perceptual indices of sensory attenuation relate. To do so, using Electro Encephalography (EEG), we adapted a luminance discrimination protocol that has been used before successfully to assess perceptual sensitivity and response bias of anticipated and unanticipated visual action effects (Roussel et al., 2013). Roussel et al. made participants learn an association between left and right key presses and the presentation of the letters A and H, respectively. They then made participants perform left and right key presses that randomly triggered presentation of either an H or and A at one of two possible contrasts. Participants were required to make discrimination judgment between the two contrasts. They showed contrast discrimination to be worse when the prediction (H or A, as learned during the association phase of the experiment) matches the true stimulus. Importantly, this paradigm does not only manipulate whether an action effect is predicted or not, but also the action effects’ physical energy (contrast, as we used visual stimuli). It, thus, allows us to test whether or not prediction influences the same early components in the EEG as physical stimulus energy. If this is the case, then the effect of prediction (sensory attenuation) is likely an early, low-level phenomenon. Moreover, assessing both psychophysical and neurophysiological measures of sensory attenuation enables us to tell how neurophysiological components and reduced awareness of the action effects interrelate.

## Materials and methods

### Stimuli

The stimuli were two white letters (A and H) presented within a virtual square of 3.3° of visual angle and displayed on a 24 inch LED monitor at a 60 Hz refresh. These two stimuli were presented at two different contrast values (C0 and C1, determined for each subject; see Section Contrast Determination Phase) at the center of the screen. In the test phase we used a uniform noise texture to increase perceptual variance. This noise texture was re-sampled on each screen refresh with always the same number of white and black pixels. The mean luminance of the noise was equal to the gray background.

### Contrast determination phase

In order to determine individual contrast values C0 and C1 yielding a discrimination d′ of about 1.5, every participant completed a psychophysical staircase converging on 90% correct responses in a letter identification task (A vs. H). This correct response rate was used to ensure that the stimuli were supraliminal and that we could then independently manipulate discrimination. We used the resulting contrast value as the referential contrast C0 in a Two Alternative Forced Choice (2AFC) paradigm (with constant stimuli ranging from C0 to C0 + 12%) in order to calculate the contrast value of C1 yielding 80% correct responses in a luminance discrimination task (C0 vs. C1). For an ideal observer, this contrast yields a discrimination d′ of around 1.5 (Macmillan and Creelman, 1991). This phase lasted on average 5 min.

### Association phase

Participants fixated on a 3.3° visual angle square located at the center of the screen. They were asked to press with their right/left index finger one of two keys (right and left on a response pad), each key press triggering presentation of a visual effect (A or H). The key-letter mapping was counterbalanced across participants. The letters appeared 200 ms after the key press at full contrast in the square at the center of the screen.

There were two types of association phase. First, in the free association blocks (“FreeAsso”) a sequence of 50 actions (left/right) was freely generated by the participants at a pace of about 1 key press per second. The experimenter demonstrated the pace to the participants before the experiment. We also measured the time participants needed for each block to control their pace. In 5% of the trials the visual effect was a W. In these catch trials, the participant had to press both buttons within 1 s of the appearance of the stimulus. Catch trials were meant to ensure that participants paid attention to the effect stimuli. Second, in memory association blocks (“MemoryAsso”), random lists of As and Hs were presented to the participants (the average list size was 5 going from 3 to 8 items adapted to the participants performance with a simple 1up 1down rule). The lists were presented via headphones as spoken letters. After the lists were presented, participants had to reproduce the sequence by pressing the corresponding button sequence. This phase was meant to foster the learning of the action-effect associations. We reasoned that when participants have to generate an action given a desired outcome, the action-effect relationship will be encoded particularly strongly.

The association phase consisted of three FreeAsso blocks and two MemoryAsso blocks. Each FreeAsso block contained 50 trials. Each MemoryAsso block contained 30 sequences of, on average, 5 items. Each Participant ran three FreeAsso and two MemoryAsso blocks. This phase took on average 25 min.

### Test Phase

Participants fixated at a square at the center of the screen, just as in the association phase. They were asked to produce, at random, right and left key presses. Again, the key presses triggered presentation of letter stimuli 200 ms after the key press. In this phase, however, Hs and As were presented randomly after each key press, such that 47.5% of the generated stimuli were congruent with the previous association (i.e., the letter corresponded to the one associated to that key press in the association phase), and 47.5% were incongruent. On the remaining 5% of trials, no stimulus was presented. The stimuli appeared randomly (but in equal proportions) with the luminance C0 or the luminance C1. Participants were told that there were two categories of luminance ranging from the value 0–49 for the C0 category and from 51 to 100 for the C1 category. In order to maintain this uncertainty about the contrast on 5% of trials stimuli appeared with a random contrast between C0 − 15% contrast and C1 + 15% contrast. After the stimulus had disappeared participants were required to judge the luminance value of the stimulus on a luminance response bar. On this bar participants could place the cursor on the perceived contrast value with values of 49 and under corresponding to C0 and 51 and over corresponding to C1. Participants completed 3 tests blocks of 44 trials (on average 25 min) before running a re-association phase composed of one of each type of association block (10 min on average). Thereafter, they ran another tests blocks. In total participants responded to 264 test trials. The experiment lasted on average 1.30 h. Participants could take short breaks prior to each of the association phases.

### Analysis of discrimination performance

The luminance discrimination task was considered to be a yes/no protocol, with C1 being the target. That is, a C1 response to a C1 stimulus is a hit, a C1 response to a C0 stimulus is a false alarm, etc. According to Signal Detection Theory (Green and Swets, 1996), d′ (perceptual sensitivity) and c (response bias) are calculated using *d′* = *z*(hit rate) − *z*(false alarm rate) and *c* = −0.5 × [*z*(hit rate) + *z*(false alarm rate)])^1^. Since participants provided their judgments of contrast using a continuous scale from 1 to 100 this allowed us to analyze not only their overall contrast judgment (C0 or C1) but also their rating of the perceived intensity (contrast) of the stimulus. We split the ratings into 10 bins to compute ROC (receiver operating characteristic) curves for each participant. The area under the curve, A′ was calculated such that A′ = 1/2*Σ(F_i+1_-F_i_)(H_i+1_+H_i_) , with *F* and *H* being False alarms and Hits respectively (Macmillan and Creelman, 1991). d′ , c and A were calculated separately for congruent and incongruent trials.

### EEG recording and data preprocessing

EEG was recorded with 64 electrodes (actiCAP, Brain ProductsGmbH, Germany). The EEG was digitized at 500 Hz. EEG analysis was conducted using EEGLAB (Delorme and Makeig, 2004) and custom-built Matlab scripts. The data were resampled offline to a 250 Hz sample rate, low band-pass filtered at 45 Hz to remove line noise. Epochs were generated from −500 to 980 ms relative to stimulus onset, with a 200 ms prestimulus baseline correction. Initial artifact rejection was conducted in a semiautomatic manner (in EEGLAB) by rejecting epochs with activity above 100 μV or below** −**100 μV, as well as rejecting trials where activity at any time point for any electrode was more than 5 standard deviations from the mean activity for that epoch. Any channels that contributed to the rejection of many epochs were considered for removal and later interpolation. Frontal channels that showed large amplitude blink activity were also excluded from further analysis during this this first semiautomatic artifact rejection. Ocular artifact correction was conducted in EEGLAB in Matlab using independent component analysis (Delorme and Makeig, 2004). Following removal of eye blinks and eye movements, noisy channels were replaced by an interpolated weighted average from surrounding electrodes. Data were then re-referenced to the common average. A final round of semiautomatic artifact rejection with a threshold of +/− 80 μV was used to remove any remaining artifacts. All ERPs are presented with a low-pass filter of 20 Hz for visual presentation purposes.

An ANOVA with the factors congruency (congruent, incongruent) and stimulus contrast (C0, C1) was run on ERPs averaged for each participant. Since our task involved visual stimuli, we focused our analysis on a region of interest at the occipital electrodes (O1 Oz O2). Since our stimuli were degraded and presented in a continuous stream of background visual noise, we postulated that this might influence the latency of the visual response. Therefore we inspected the ERPs over our region of interest to determine the time window corresponding to an apparent peak for the visual stimulus. The time window for analysis was selected around the peak of this visual component. Importantly, this time-window was not selected based on the difference between our conditions but rather on the presence of the component itself. Analysis of the modulation of this component by contrast, would then provide further justification for the time-window, since stimulus contrast should influence the magnitude of the visual response. It is important to note that the main comparison of interest—the congruency effect—was orthogonal to the contrast effect, and therefore the selection of the time-window would not unduly bias this comparison.

### Participants

Nineteen participants took part in the experiment. They were naive to the purpose of the experiment. Four of these nineteen participants were excluded from the analysis as their luminance discrimination d′ s were below 0.5 (for 2 of them) or because the ratio of right left key presses during the test phases exceeded a 75% 25% ratio (for one of them). One was rejected because of the poor quality of the EEG recordings. Seven of the remaining participants had action-effect mapping 1 (left → A, right → H), and 8 had mapping 2 (8 women, 7 men; mean age = 24 years, SEM** = 3.69 years).

## Results

In order to ensure that the data were equivalent between the EEG and the behavioral analysis only trials free from EEG artifacts were analyzed. The amount of rejected data was less than 10% of the total number of trials.

### Psychophysical Results

**Figure 1:**
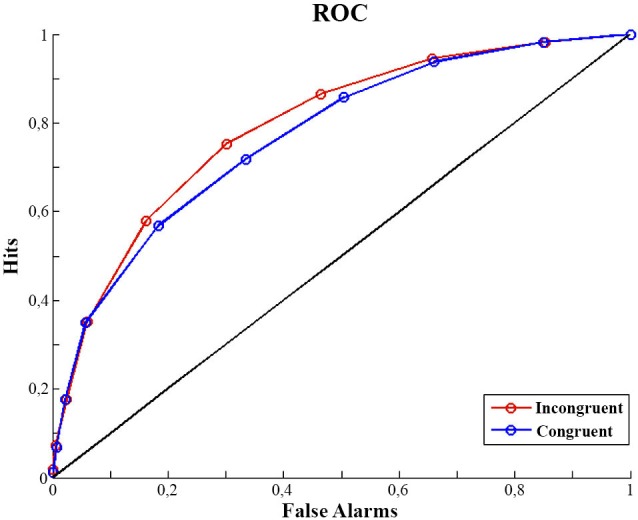
**ROC curves**. Mean of participants ROC curves for congruent and incongruent trials. For calculation detail of the ROC see Analysis of Discrimination Performance section.

We analyzed our data dependent on participants’ contrast discrimination. Discrimination performance (d′ ) was lower in the congruent condition (d′ congruent: 1.22, *SD* = 0.46) than in the incongruent (d′ incongruent: 1.42, *SD* = 0.45) condition. A one factor repeated measures analysis of variance including the factor of congruency showed this effect of congruency on d′ to be significant (*F*_(1,14)_ = 5.36, *p* = 0.03). At the same time, the criterion (c) was not different in the two congruency conditions (1 factor repeated measures ANOVA; *F*_(1,14)_ = 0.165, *p* = 0.69). A bias free measure of sensitivity (A′) also confirms our finding. Sensitivity appears to be better for incongruent trials (A′: 0.81, *SD* = 0.075) than for congruent trials (A′: 0.79, *SD* = 0.067) (*F*_(1,14)_ = 4.670, *p* = 0.04) (see Figure 1). In contrast the criterion (bias) appeared not to differ between conditions (Congruent: 0.39, *SD* = 0.46; Incongruent: 0.41, *SD* = 0.54; *F*_(1,14)_ = 0.165, *p* = 0.691).

### EEG Results

**Figure 2:**
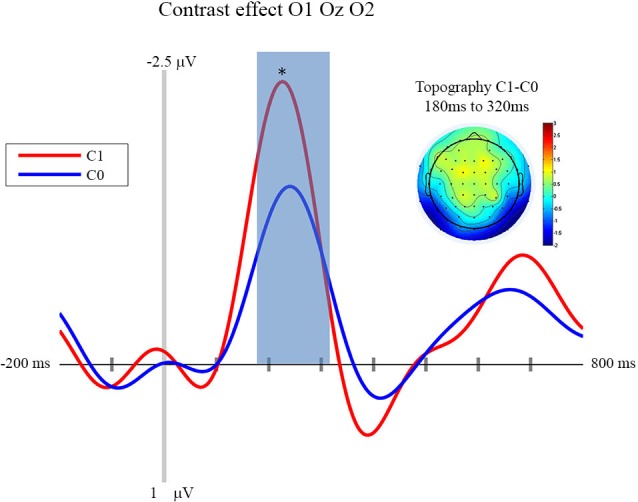
**Contrast effect: ERP and Topography**. This figure presents the mean ERPs on O1 Oz O2 for C0 (in blue) and C1 (in red) from −500 to 980 ms relative to stimulus apparition. The blued surface represents the analysis time window (from 180 to 320 ms, centered on the pic around 250 ms). In the top left corner the topography of the difference (C1 − C0) is presented for the analysis time window. * *p* < 0.05.

**Figure 3:**
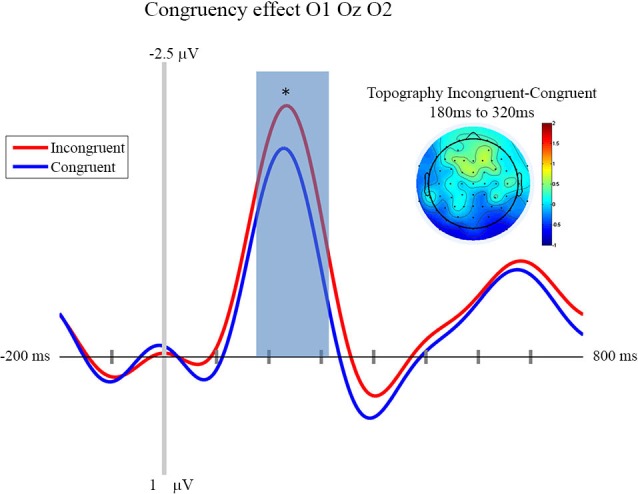
**Congruency effect: ERP and Topography**. This figure presents the mean ERPs on O1 Oz O2 for congruent (in blue) and incongruent (in red) from −500 to 980 ms relative to stimulus apparition. The blued surface represents the analysis time window (from 180 to 320 ms, centered on the pic around 250 ms). In the top left corner the topography of the difference (Incongruent—Congruent) is presented for the analysis time window. * *p* < 0.05.

In this section we focus on the effect of motor prediction on the neurophysiological indices of visual processing to determine the degree to which behavioral and neural sensory attenuation are related. The ERPs and the topographies for the different conditions are presented in Figures 2, 3. A large negative deflection is apparent in all the waveforms, peaking at around 250 ms after stimulus onset. This peak appears to be greater for C1 than C0, such that it likely reflects processing of the visual stimulus (a delayed visual N1 component) (Ciesielski and French, 1989; Mangun and Hillyard, 1991; Johannes et al., 1995). To quantify these effects we took the average amplitude of each condition in a 140 ms time window centered on this peak (180–320 ms). A repeated measure analysis of variance including the factor of contrast and congruency revealed a significant main effect of Contrast (*F*_(1,14)_ = 6.54, *p* = 0.023), confirming significantly greater amplitude for C1 (mean = −1.67; std = 1.55, CI: +/− 1.21) compared to C0 (mean = −0.81; std = 1.8, CI: +/− 1.63). The topography of this difference is consistent with modulation of an occipital ERP component. We also observed a significant main effect of Congruency (*F*_(1,14)_ = 11.36, *p* = 0.005), such that our visual component was of significantly smaller amplitude in the congruent condition (mean = −0.99; std = 1.57, CI: +/− 1.24), compared to the incongruent condition (mean = −1.50; std = 1.64, CI: +/− 1.36). The topography of this difference is also consistent with a modulation of visual processing as a function of action prediction. However the interaction between both factors was not significant (*F*_(1,14)_ = 0.02, *p* = 0.885).

In line with the aim of the current experiment, it appears that this visual ERP peaks as a function of congruent motor prediction. Since we also observed attenuated sensitivity for congruent trials in the behavioral analysis presented above, this provides evidence that neural and behavioral measures of sensory attenuation are likely related.

## Discussion

First of all, our experiment shows that neurophysiological sensory attenuation is a phenomenon that is not restricted to the auditory and somatosensory modality, but that it can also be observed in the visual domain. More importantly, one of the aims of the experiment presented above was to investigate the locus of sensory attenuation. This was done by way of comparing the influence of prediction and stimulus contrast, respectively, on the ERPs triggered by visual action effects. We observed that an N1 component was clearly modulated by stimulus contrast, with larger contrasts resulting in a larger deflection. Importantly, congruency affected the very same component, with congruent trials resulting in smaller amplitudes than incongruent trials.

Of course, our data cannot show which processing stage precisely the two factors manipulated in the current experiment influence. The current paradigm differs in important aspects from other studies investigating visual evoked potentials. Notably, we presented stimuli in visual noise resampled at each screen refresh. The actions’ effects were, therefore, not presented with a sharp onset. Moreover, in our experiment, stimuli were triggered by an action. It is therefore difficult to compare our results to ERPs found in previous studies. However, previous research seems to suggest that contrast-dependent processes take place rather early in the visual processing stream (e.g., Schadow et al., 2007), while later components are rather modulated by motion and form perception (Bach and Ullrich, 1997; Göpfert et al., 1998). Importantly, our experiment allowed us to directly compare the effect of contrast and prediction. As it demonstrates that motor prediction influences the same processing stage as visual contrast, we assume that motor prediction as manipulated in our experiment influences an early processing stage that is otherwise still modulated by basic stimulus-features. This interpretation is corroborated by the fact that the N1 component in question has an occipital topography. That this component has a relatively late latency is probably due to the fact that the stimuli used in the current experiment were not presented with a sharp onset, but embedded in dynamic pixel noise, such that the detection of a pattern is more time-consuming.

This evidence of reafferent attenuation in the visual modality is also important regarding to the literature of saccadic suppression (Bridgeman et al., 1975; Deubel et al., 1996) thought to rely on a “corollary discharge” from the motor command affecting the perceptual network (Sperry, 1950; Paus et al., 1995). Nonetheless the link between sensory attenuation and saccadic suppression must be more thoroughly investigated since some essential differences separate both phenomena, notably the timing of the effect. For example sensory attenuation has been shown to occur on the final consequence of the action (Blakemore et al., 1998) while saccadic suppression has been shown to occur during the saccade (Bridgeman et al., 1975; Deubel et al., 1996).

The second aim of the present study was to explore how neurophysiological and perceptual indices of sensory attenuation relate. We used a luminance discrimination protocol to assess perceptual sensitivity and response bias of anticipated and unanticipated visual action effects, assessing EEG activity at the same time. The psychophysical results show that discrimination performance (d′, A′) was better in the incongruent condition than in the congruent condition. At the same time, the criterion was not different in the two congruency conditions. The results, thus, are in line with the findings of Roussel et al. (2013) and Cardoso-Leite et al. (2010). They show that contrast sensitivity is reduced when a motor act provides an accurate prediction of the ensuing visual stimulus.

As concerns the effect of congruency on ERPs, we observed that the contrast-sensitive visual component was significantly smaller in the congruent condition compared to the incongruent condition. We, thus, observed, to our knowledge for the first time, sensory attenuation in psychophysical and neurophysiological indices at the same time, suggesting that the two measures of sensory attenuation are likely related. However, note that the psychophysical effect corresponds to an interaction between contrast and congruency: the discrimination between the two contrast levels is more difficult in congruent than in incongruent trials. If sensory attenuation assessed with psychophysical methods were a direct reflection of the ERPs assessed at the same time, we would have expected to see an interaction between these two factors in our ERP data as well. However, this was not the case. Of course, it might be that the ERP data simply lack sufficient statistical power. However, it is also possible that psychophysical and ERP indices of sensory attenuation (at least those assessed in the current experiment) are not in a simple one-to-one relationship. Perception might be dependent not only on early cortical responses, but also on later processing and/or recurrent processing, tweaking the relationship between perceptual measures and observable neurophysiological measures.

In conclusion, in our experiment ERP effects of visual sensory attenuation were found to correspond to contrast-dependent processing stages. We conclude that motor prediction, thus, influences quite early processes. Moreover, we demonstrated that both psychophysical and ERP indices of sensory attenuation can be observed in the visual modality. However, the exact relationship between the two types of measure needs to be further clarified, as there are not only commonalities, but also differences.

## Conflict of interest statement

The authors declare that the research was conducted in the absence of any commercial or financial relationships that could be construed as a potential conflict of interest.
